# Evaluation of Knowledge About Gestational Diabetes Among Primigravidae Versus Multigravidae in Saudi Arabia: A Quantitative Cross-Sectional Study

**DOI:** 10.7759/cureus.50458

**Published:** 2023-12-13

**Authors:** Albagir M Hassan, Ghadi S Alghamdi, Abdulrahman M Alfantoukh, Ajyal Aljohani, Fahad A Alzahrani, Ghaida A. Eissa, Lama A Alkhedewi, Lubna Aloufi, Ola A Khawaji, Rahaf Khurmi

**Affiliations:** 1 Obstetrics and Gynaecology, Umm Al-Qura University, Makkah, SAU; 2 Faculty of Medicine, Albaha University, Al Baha, SAU; 3 Faculty of Medicine, Al-Imam Muhammad Ibn Saud Islamic University, Riyadh, SAU; 4 Faculty of Medicine, Taibah University, Medina, SAU; 5 Faculty of Medicine, Bisha University, Bisha, SAU; 6 Faculty of Medicine, King Abdulaziz University Faculty of Medicine, Jeddah, SAU; 7 Faculty of Medicine, Princess Norah Bint Abdulrahman University, Riyadh, SAU; 8 Faculty of Medicine, Jazan University, Jazan, SAU

**Keywords:** mecca, makkah, antenatal education, pregnancy, gestational diabetes mellitus, saudi women knowledge, risk factors of gdm, management of gdm

## Abstract

Background: Gestational diabetes mellitus (GDM) is a subtype of diabetes that is discovered during pregnancy and may disappear after puerperium. It has an impact on the well-being of expectant mothers and fetuses. Many women have a poor level of knowledge regarding GDM, especially regarding risk factors, diagnosis, and the role of insulin in the management. Also, knowledge, attitudes, and practices regarding GDM are influenced by multiple factors.

Objectives: The objective of this study was to evaluate the level of knowledge among primigravidae versus multigravidas regarding GDM. Additionally, the study sought to identify the factors that are associated with high and low levels of knowledge regarding GDM.

Methodology: This is a prospective, cross-sectional study, conducted in different regions of Saudi Arabia and included 482 women who had a pregnancy history or were currently pregnant. Data were collected using a questionnaire consisting of two parts. A pilot study was carried out to evaluate the validity of the questionnaire.

Results: The study data showed that a substantial portion of respondents have had one pregnancy, while there is a relatively even distribution among those with two (n=89, 18.50%), three (n=75, 15.60%), or four or more pregnancies (n=71, 14.70%). Of the participants, 65.6% had good knowledge regarding GDM. It was also found that there were significant differences between primigravidae and multigravidae.

Conclusion: One-third of the participants in both groups had poor knowledge with significant variation in knowledge between primigravidae and multigravidae. The importance of screening and fasting before testing received widespread recognition, as did the role of diet and exercise in treatment. It is recommended that targeted educational efforts and awareness campaigns be launched to enhance knowledge about GDM among pregnant women in Saudi Arabia.

## Introduction

Gestational diabetes mellitus (GDM) is a subtype of diabetes mellitus [1]. It is a frequent complication that affects the health of pregnant women and fetuses, making pregnancy high‑risk [1,2]. It has a high prevalence in Saudi Arabia [3]. Gestational diabetes is linked to perinatal maternal and infantile adverse outcomes; maternal outcomes in women with GDM have a higher rate of labor induction and C-section delivery in comparison to non-GDM mothers, and infants of mothers with GDM were linked with a higher rate of lower APGAR score, more neonatal intensive care unit (NICU) admission, and hypoglycemia compared to infants of mothers without GDM [3]. A study conducted in a primary healthcare center in Saudi Arabia found that more than half of the women assessed had a poor level of knowledge regarding GDM, especially regarding risk factors, diagnosis, and the role of insulin in the management [3]. A higher level of knowledge was recorded in women who lived in urban areas, had a higher level of education, were younger, or had somebody in their family working in the medical field [3].

A cross-sectional study in Zambia explored how educational level, social status, gravidity, and parity influenced awareness, knowledge, attitude, and practices regarding GDM among women attending antenatal clinics in Mufulira Town, Zambia [4]. The study indicated poor level of knowledge about GDM and the lack of educational programs on diabetes in pregnancy was deduced to be a contributing factor. Therefore, the way forward is to set up a structured educational program on GDM and its complications as a component of antenatal and child health clinics [4]. Moreover, a study done in Bangladesh found that the majority of the participants had poor knowledge but showed a positive attitude regarding GDM control and the GDM education program [5]. A qualitative study in Vietnam published in 2012 showed that women were concerned about the transmission of GDM through breast milk and planned not to breastfeed [6]. The study highlighted the need for culturally appropriate clinical education and health promotion activities for women with GDM. Another study conducted in Samoa found that most participants were unaware of GDM and its potential consequences [7]. In this, only a minority of women correctly identified all risk factors for GDM, despite recognizing the importance of a healthy diet and physical activity in preventing GDM.

There is a lack of studies regarding the level of knowledge of GDM in Saudi Arabia, even though it is a common health issue faced by pregnant women in the region. Hence, this study aims to evaluate the knowledge of primigravidae and multigravidae and determine factors linked to higher and lower levels of knowledge among Saudi women.

## Materials and methods

Study design and sample technique

This study was conducted using a prospective, cross-sectional design in Saudi Arabia, from August to October 2023. All Saudi pregnant women were included in the study while non-Saudi women, incomplete responses, and those who refused to participate in the study were excluded. We divided Saudi Arabia into five regions (Northern Region, Southern Region, Eastern Region, Western Region, and Central Region). The subjects were chosen using the cluster random sampling technique. The questionnaire was self-administered and included all who met the inclusion criteria. The distribution of the sample size by region was calculated according to the population size of each. The sample size calculated for this study was 482 participants. The selection was made using a convenience sampling technique, with a 95% confidence level and a 5% margin of error. The calculations were made using the online Raosoft sample size calculator [8].

Data collection

The data were collected using an original questionnaire, which was sent via social media platforms to the targeted sample. The questionnaire included two parts. The first section included questions on sociodemographic information, which included age, region of residence, educational level, current employment status, order of pregnancy, family history of GDM, and personal history of GDM. The second section included questions to determine knowledge about GDM risk factors, screening, treatment options, and complications. The Answers to these questions were designed as two choices Likert scale (agree, disagree, and I don’t know). A score of 75% and above right answers was defined as good knowledge, 55-74% was average, and below 54% was poor.

Validity and analysis

A pilot study was carried out to evaluate the validity of the questionnaire, which was used in the study for data collecting, and accordingly, the necessary modifications were made if any. The design used for the pilot test was descriptive; a questionnaire was filled by participants obtained from 15 women and the result was not included in the final report, Cronbach’s alpha (0.862) was used. The sample of participants was selected for the pilot test from the same population to be considered for the main study. After data collection, the data were analyzed by using IBM SPSS Statistics for Windows, Version 22.0 (Released 2013; IBM Corp., Armonk, New York, United States). Data were analyzed using several tests, which include the Chi-square test, T-test, and Pearson Correlation coefficient test.

Ethics and confidentiality

This cross-sectional study was authorized by the Biomedical Research Ethics Committee at the Umm Al-Qura University in Makkah, Saudi Arabia. The questionnaire's anonymity was chosen to maintain the privacy of the participant's responses.

## Results

Table 1 presents a comprehensive overview of participant demographics across four variables (age, region, education, employment). A total of 482 women were included in this study. Almost three-fourths of the respondents fall within the 20-30-year age group, accounting for 72.8% (n=351) of the total. The highest number of participants came from the East region, comprising 28.6% of the total sample, while the North and South regions had the fewest participants. Approximately three-fifths of the respondents had a high level of education (Bachelor, Master, or PhD), making up 59.8% (n=288) of the sample. A significant portion of the respondents (n=260, 53.9%) were unemployed, while 28.2% (n=136) were employed. Additionally, there was a small percentage (n=9, 1.9%) of retired individuals and students.

**Table 1:** Demographic Characteristics of Study Participants, n= 482

Studied variable		Number	Percentage
Age	Less than 20 years	30	6.2
	20-25 years	160	33.2
	25-30 years	191	39.6
	More than 35 years	101	21.0
Region	Central	109	22.6
	East	138	28.6
	North	57	11.8
	South	46	9.5
	West	132	27.4
Educational level	Diploma	59	12.2
	Bachelor, Master, PhD	288	59.8
	High school	106	22.0
	Illiterate	6	1.2
	Primary/Middle school	23	4.8
Current employment status	Employed	136	28.2
	Unemployed	260	53.9
	Student	77	16.0
	Retired	9	1.9

**Figure 1:**
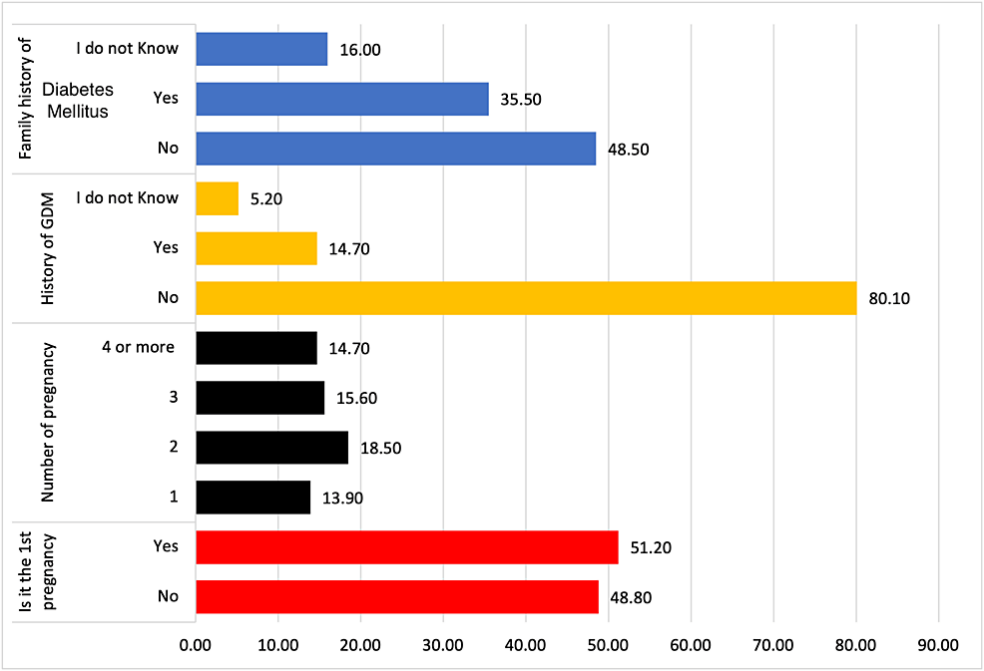
Pregnancy and Diabetes-Related Variables: First Pregnancy, Number of Pregnancies, History of GDM, and Family History of Diabetes Mellitus Data Given as Percentage (number values given in the text) GDM, Gestational Diabetes Mellitus

Figure 1 shows a comprehensive overview of various variables related to pregnancy and diabetes, with information on the number and percentage of participants that fall into different categories for each variable. Regarding the variable "Is it your 1st pregnancy," there was a relatively balanced distribution between "No" (n=235, 48.80%) and "Yes" (n=247, 51.20%). When considering the "Number of pregnancies," the data shows that a substantial portion of respondents had one pregnancy (n=247, 13.90%), while there is a relatively even distribution among those with two (n=89, 18.50%), three (n=75, 15.60%), or four or more pregnancies (n=71, 14.70%). The category "History of GDM" illustrates a lower prevalence of GDM (n=71, 14.70%), but is notable, and a small percentage (n=25, 5.20%) expresses uncertainty about their GDM history. The variable "Family history of diabetes mellitus" displays a fairly even split between those with (n=171, 35.50%) and without (n=234, 48.50%) a family history of diabetes mellitus, with 77 (16.00%) indicating they did not know their family history. 

Table 2 provides insights into the knowledge and perceptions of the respondents on various aspects of gestational diabetes (GDM). Notably, the majority of participants agreed that family history can lead to GDM (n=344, 71.5%) and that obesity is a potential cause (n=331, 68.8%). Additionally, a significant portion recognized that GDM may result from a prior history of GDM (n=333, 69.2%). The belief in the importance of screening all antenatal women for GDM was strong (n=431, 89.6%), and a significant proportion acknowledged the need to fast before screening (n=348, 72.3%). Regarding treatment, most respondents believe that diet and exercise are part of the treatment plan (n=402, 83.6%), while opinions are more divided on the use of insulin (n=244, 50.7%) and oral medications (n=259, 53.8%). Moreover, participants were generally uncertain about the effects of GDM on abortions (n=166, 34.5%) and the possibility of it causing congenital anomalies (n=162, 33.7%). There was recognition that GDM can affect the baby's size (n=347, 72.1%), lead to preterm delivery (n=339, 70.5%), and cause neonatal problems (n=289, 60.1%). A significant proportion also understands that GDM may not necessarily disappear after delivery (n=303, 63.0%) and could potentially develop into common diabetes (n=328, 68.2%). 

**Table 2:** Level of Knowledge Across the Surveyed Population GDM, Gestational Diabetes Mellitus

Knowledge		Number	Percentage
Is it possible to develop GDM if there is a family history of GDM?	Agree	344	71.5
	Disagree	52	10.8
	I do not Know	86	17.9
Is obesity a cause of GDM?	Agree	331	68.8
	Disagree	85	17.7
	I don’t know	66	13.7
Is it possible to develop GDM if there is a previous GDM?	Agree	333	69.2
	Disagree	62	12.9
	I don’t know	87	18.1
Is it important to screen all antenatal women for GDM?	Agree	431	89.6
	Disagree	17	3.5
	I don’t know	34	7.1
Is it important to be fasting before the screening?	Agree	348	72.3
	Disagree	38	7.9
	I dont know	96	20.0
Is diet and exercise part of the treatment plan for GDM?	Agree	402	83.6
	Disagree	18	3.7
	I don’t know	62	12.9
Is insulin part of the treatment plan for GDM?	Agree	244	50.7
	Disagree	97	20.2
	I don’t know	141	29.3
Is oral medications part of the treatment plan for GDM?	Agree	259	53.8
	Disagree	65	13.5
	I don’t know	158	32.8
Does GDM have an effect on Abortions?	Agree	249	51.8
	Disagree	67	13.9
	I don’t know	166	34.5
Is GDM a cause of congenital anomalies?	Agree	215	44.7
	Disagree	105	21.8
	I don’t know	162	33.7
Does GDM affect the size of the baby?	Agree	347	72.1
	Disagree	30	6.2
	I don’t know	105	21.8
Is preterm delivery a complication of GDM?	Agree	339	70.5
	Disagree	32	6.7
	I don’t know	111	23.1
Is neonatal problems a complication of GDM?	Agree	289	60.1
	Disagree	52	10.8
	I don’t know	141	29.3
Does GDM disappear after delivery?	Agree	303	63.0
	Disagree	73	15.2
	I don’t know	106	22.0
Is it possible for GDM to develop into common diabetes?	Agree	328	68.2
	Disagree	59	12.3
	I don’t know	95	19.8

Table 3 shows that, in general, 34.4% (N=166) of the participants had poor general knowledge. Knowledge levels vary among respondents based on their demographic characteristics. There were no significant differences in knowledge levels between different age groups. Knowledge levels vary significantly by region, with the Eastern region having significantly better knowledge compared to the other regions (p=0.004). There was a difference in knowledge levels between educational levels; however, this difference was not statistically significant (p=0.069). Employment status was a significant factor affecting knowledge, with employed and retired individuals having significantly better knowledge compared to others (p=0.001). Knowledge level did not differ significantly between those with and without a previous pregnancy (p=0.255) suggesting that having a previous pregnancy does not have a strong impact on knowledge of GDM. Although there was a noticeable difference in knowledge levels between those with and without a family history of GDM, (p=0.204), this difference was not statistically significant. Finally, knowledge levels did not vary significantly based on a previous history of GDM (p= 0.561).

**Table 3:** Knowledge of GDM Across Demographic Variables: An Analysis of Age, Residence, Education, Employment, Pregnancy History, Family History, and Previous GDM History X2: chi square test; p <0.05 was considered significant GDM, Gestational Diabetes Mellitus

Variables		Poor knowledge (n = 166)		Good knowledge (n = 316)		Test	p-value
		Number	Percentage	Number	Percentage		
Age	Less than 20 years	11	36.67	19	63.33	1.34	0.721
	20-25 years	58	36.25	102	63.75		
	25-30 years	67	35.08	124	64.92		
	More than 35 years	30	29.70	71	70.30		
Residence	Central region	40	36.70	69	63.30	15.64	0.004
	Eastern region	34	24.64	104	75.36		
	Northern region	30	52.63	27	47.37		
	Southern region	19	41.30	27	58.70		
	Western region	43	32.58	89	67.42		
Education	Diploma	21	35.59	38	64.41	8.71	0.069
	High level education	90	31.25	198	68.75		
	High school	39	36.79	67	63.21		
	Primary/Middle school	14	60.87	9	39.13		
	Illiterate	2	33.33	4	66.67		
Current employment status	Employed	32	23.53	104	76.47	16.07	0.001
	Unemployed	110	42.31	150	57.69		
	Student	22	28.57	55	71.43		
	Retired	2	22.22	7	77.78		
Is this your first pregnancy?	No	75	31.91	160	68.09	1.30	0.255
	Yes	91	36.84	156	63.16		
Do you have family history of GDM?	No	87	37.18	147	62.82	3.18	0.204
	Yes	50	29.24	121	70.76		
	I do not know	29	37.66	48	62.34		
Do you have a previous history of GDM?	No	132	34.20	254	65.80	1.15	0.561
	Yes	23	32.39	48	67.61		
	I do not know	11	44.00	14	56.00		

There were significant differences between primigravidae and multigravidae in the following knowledge: the possibility of developing GDM if there is a family history of GDM (p=0.046); obesity could cause GDM (p=0.031); the importance of screening all antenatal women for GDM (p<0.001); the role of diet and exercise in the treatment of GDM (p=0.005); the potential effect of GDM on the size of the baby (p=0.032); the association between GDM and preterm delivery (p=0.028); association between neonatal problems and GDM (p=0.004). In general, multigravidae had a better level of knowledge than primigravidae even though the latter were more in our sample than the former.

## Discussion

The global prevalence of type 2 diabetes is increasing, especially in Saudi Arabia [9], and this rise is mirrored by an increase in GDM. To facilitate early diagnosis of GDM, expectant mothers must be well-informed about the risk factors, screening methods, and potential complications [10]. The main objectives of the current study include two critical aspects of GDM knowledge: first, discern differences in awareness between first-time pregnant women (primigravidae) and those with multiple pregnancies (multigravidae), shedding light on potential disparities that could inform customized educational interventions. Second, the study aims to pinpoint the factors that contribute to the various levels of knowledge about GDM among the participants.

Regarding the level of knowledge, 34.4% (N=166) of the respondents had poor knowledge. There were statistically significant differences between primigravidae versus multigravidae in the following knowledge: the possibility of developing GDM if there was a family history of GDM, obesitycan cause GDM, the importance of screening all antenatal women for GDM, the role of diet and exercise in treatment, the potential effect of GDM on the size of the baby, the association between GDM and preterm delivery, and the association between neonatal problems and GDM.

In the current study, it is noteworthy that 316 (65.6%) had good knowledge about GDM. Inversely, a cross-sectional study conducted in Almadinah Almunawarah, Saudi Arabia, among women attending primary healthcare centers found that over half of the participants (n=178, 53.45%) had a poor level of knowledge about GDM, while only 7.80% (N=26) possessed a good level of knowledge on the subject [11]. Similarly, Kondamuri et al. reported that the majority had a low level of knowledge and 51.5% (N=103) of participants demonstrated a poor level of knowledge regarding GDM, whereas a smaller proportion, specifically 14.5% (N=29), exhibited a deficiency in information regarding GDM [10]. This was somewhat different from the findings of other studies. For example, the study by Dhyani et al. reported that more than half of women (57.6%) had an average level of knowledge about GDM, with 21.8% showing good knowledge and 19% displaying poor knowledge [12]. A study conducted by Mahalakshmi et al. found that only 35.2% of participants had good knowledge about GDM [13]. Similarly, in a study conducted in Bangladesh, it was revealed that 26.3% of the participants had a good level of knowledge about GDM [14]. The variations in knowledge levels observed across different studies may be attributed to differences in study settings and the assessment tools employed. Additionally, the choice of sampling techniques, whether random or non-random, could also play a significant role in these variations.

Moreover, our study revealed significant associations between residence and employment status with the level of knowledge of GDM. In contrast, factors such as education, age, and the number of pregnancies did not exhibit any statistically significant associations with knowledge level. In previous studies, factors such as age [12], education [9,12,13,15,16], and residence (urban vs. rural) [13,15-17] were reported to be associated with knowledge level. Indeed, the place of residence and employment can significantly impact the level of knowledge, as it may be associated with access to health services and interactions with other individuals who can serve as important sources of information. These findings underscore the importance of considering specific demographic variables, such as residence and employment status, when tailoring educational interventions to improve awareness and understanding of GDM among the study population.

As for the comparison between multigravidae and primigravidae, the current study found a statistically significant difference in the knowledge level. Similarly, Thomas et al. reported that the number of pregnancies emerged as a significant predictor of awareness, with an odds ratio of 0.35 [18]. This suggests that respondents with a higher number of pregnancies were 0.35 times more likely to report awareness compared to those with fewer gestations. Likewise, Dissassa et al. reported that women who had experienced previous pregnancies (multigravidae) were found to be three times more likely to have adequate knowledge of GDM compared to women who were pregnant for the first time (primigravidae) [19]. We believe that undergoing pregnancy more than one time, which may make the patient exposed to a larger number of doctors and nurses, may play an important role in increasing information about the condition and its complications, risk factors, and preventive methods.

Strengths and limitations

This prospective cross-sectional study had a range of strengths and limitations worth noting. The study benefits from a substantial sample size, starting at 384 participants and eventually expanding to 480, which greatly enhances the statistical robustness of the study and the applicability of its findings. The use of cluster random sampling allows for the diverse inclusion of pregnant women in various regions, thus strengthening the external validity of the results. Furthermore, the pilot study and the adoption of a validated questionnaire contribute to the reliability and credibility of the data collected. However, it is important to acknowledge that due to its cross-sectional design, the study is limited in its capacity to establish causal relationships over time. Furthermore, certain limitations are associated with the survey, including the potential for recall bias. Finally, we did not ask about the source of information on GDM, which is considered an important determinant of the level of knowledge, which could affect knowledge level.

## Conclusions

This study offers valuable insights into the knowledge and perceptions of GDM among the participants. Nearly one-third of the participants had poor knowledge with significant variation in knowledge between primagravidae and multigravidae. In particular, respondents demonstrated awareness of key aspects, including the influence of family history, obesity, and previous GDM. The importance of screening and fasting before testing was well recognized, as was the role of diet and exercise in treatment. While opinions varied regarding the use of insulin and oral medications, uncertainty persisted regarding the potential impact on abortions and congenital anomalies. Furthermore, the study highlighted significant differences between primigravidae and multigravidae in their knowledge, emphasizing the importance of considering gravidity when addressing knowledge disparities.

It is recommended that targeted educational efforts and awareness campaigns be launched to enhance knowledge about GDM among pregnant women in Saudi Arabia. These initiatives should emphasize risk factors, the importance of early screening, and the role of a healthy lifestyle in the management of the condition. Tailoring interventions to specific demographic groups, such as primigravidae and multigravidae, is essential. Long-term follow-up and postnatal education for women diagnosed with GDM are crucial for effective management. To comprehend GDM-related concepts, it is important to look into the effects of residency and occupation on GDM knowledge as well as the differences between primigravidae and multigravidae. The usefulness of prenatal screening initiatives and treatments encouraging lifestyle changes for the management and prevention of GDM should also be the subject of future research. Studies with a longitudinal design can shed light on how our understanding of GDM is changing and what that means. 
